# Genome-Wide Identification and Expression Analysis of the FAR1-RELATED SEQUENCE (FRS) Gene Family in Grape (*Vitis vinifera* L.)

**DOI:** 10.3390/ijms26104675

**Published:** 2025-05-14

**Authors:** Heng Yao, Yanyan Zheng, Yanzhao Sun, Yang Liu

**Affiliations:** College of Horticulture, China Agricultural University, Beijing 100193, China; heng1105@cau.edu.cn (H.Y.); yyzyy@cau.edu.cn (Y.Z.); syzheart@163.com (Y.S.)

**Keywords:** grape, FRS gene family, phylogenetic analysis, expression pattern, stress response

## Abstract

The FAR1-RELATED SEQUENCE (FRS) family consists of transcription factors derived from transposases, playing crucial roles in plants by mediating processes such as light signaling transduction, hormone response and stress resistance. Despite the ecological and economic importance of grapes, systematic research on FRS genes in this species is limited. In this study, we identified 43 *VvFRS* genes, distributed across 13 chromosomes in grape. Phylogenetic analysis was conducted on the VvFRS family members along with Arabidopsis, tomato, and strawberry, resulting in their classification into seven distinct subgroups. Our predictions indicated that most VvFRSs were localized within the nucleus, with predominant secondary structures of α-helices and random coils. Additionally, it was observed that genes within the same subgroup exhibited a similar distribution of conserved motifs. The promoter regions of the *VvFRS* gene family harbored multiple cis-elements associated with abiotic stress, hormone response, and light signaling pathways. The RT-qPCR results revealed that the expression levels of genes in subgroups I and IV exhibited the highest abundance in leaves. Certain *VvFRS* genes demonstrated significant responsiveness to salt stress and jasmonic acid treatment. This study presents the first comprehensive identification of FRS genes in grape, offers a foundation for further functional studies.

## 1. Introduction

Light is a key environmental factor that influences plant growth and can regulate various aspects of plant development. In order to sense constant changes in light, higher plants have gradually evolved complex photoreceptor systems. At least five sets of photoreceptors are used to sense light conditions, photoperiod, ambient temperature, pathogens, and other environmental information [1,2]. Among them, phytochromes perceive red and far-red light; cryptochromes, phototropins, and zeitlupe family proteins can be used to receive UV-A radiation and perceive blue and green light; and UV RESISTANCE LOCUS 8 (UVR8) proteins can act as UV-B receptors [3,4]. Phytochromes are the main photoreceptors formed by plants for sensing terrestrial light, and they play crucial roles in seed germination, seedling formation, stem elongation, branching, circadian rhythms, and flowering time [5,6]. In Arabidopsis, five phytochromes (phyA to phyE) are encoded, of which phyA primarily mediates plants’ recognition of far-red light. Upon light activation, phyA rapidly translocates to the nucleus to initiate far-red light signaling [7]. Previous studies have shown that the nuclear translocation of phyA requires two chaperone proteins, FAR-RED ELONGATED HYPOCOTYL 1 (FHY1) and FHY1-like (FHL). Meanwhile, the transcriptional activation of FHY1 and FHL requires two more transcription factors, FAR-RED ELONGATED HYPOCOTYL 3 (FHY3) and FAR-RED-IMPAIRED RESPONSE1 (FAR1) [8].

FHY3 and FAR1 are transcription factors derived from Mutator-like element (MULE) transposases [9]. Most FAR1-RELATED SEQUENCE (FRS) family members share a conserved structure, including an N-terminal C2H2 zinc-finger domain (also known as the FAR1 DNA-binding domain), a central putative transposase domain similar to MULE transposases, and a C-terminal SWIM (SWI2/SNF2 and MuDR transposases) zinc-finger domain [10]. In Arabidopsis, twelve FRS and four FRS-RELATED FACTOR (FRF) family proteins have been identified. The FHY3/FAR1 protein exhibits transcriptional activation activity and can directly bind to the FBS site (FHY3/FAR1-binding site, CACGCGC) in gene promoters. They have evolved diverse and powerful physiological functions during plant domestication, including light signal transduction and photomorphogenesis [8,9,10,11], chloroplast division [12,13,14], chlorophyll biosynthesis [15,16], starch synthesis and starch granule formation [17], circadian clock and flowering time regulation [18,19,20,21], shoot apical meristem and floral development [22,23], oxidative stress and plant immunity [24,25], abscisic acid signal transduction and stress responses [26], nutrient absorption, and so on [27].

The FRS family members have been extensively investigated in numerous higher plants due to their crucial roles in plant growth and development, including Arabidopsis [10], Eucalyptus [28], tea plant [29], walnut [30], potato [31], and cucumber [32]. However, there is currently a lack of research on the FRS family in grape. Grape is a perennial woody vine with significant ecological and economic value [33,34,35]. It boasts a wide range of varieties and can be processed in various ways, including consumption as fresh fruit, in the production of wine, drying for raisins, or extraction for juice [36]. Consequently, it has gained global popularity through extensive cultivation. The grape cultivation process, however, exposes grapes to various forms of adversity stress such as high temperature, drought, cold, and salinity, which significantly impact the quality and economic viability of grapes [37,38,39,40,41]. The members of the FRS family play a pivotal role in plants, making it highly significant to explore and analyze the genes associated with FRS during grape development and under stress conditions.

The present study combined bioinformatics and real-time fluorescence quantitative PCR (RT-qPCR) techniques to identify members of the grape *VvFRS* gene family. The proteins were analyzed for their physicochemical properties, evolutionary relationships, conserved structural domains, interaction relations, and expression patterns. These findings aim to systematically identify and evolutionary analyze the members of the *VvFRS* family in grapes, investigate the interaction networks and regulatory pathways of VvFRS proteins, and further focus on the functional characterization and stress response mechanisms. It is anticipated that these efforts will address the current research gap regarding the FRS family in grapes, serve as a reference for other genomic analysis studies, and provide a theoretical foundation for stress-resistant breeding and fruit quality enhancement.

## 2. Results

### 2.1. Identification of VvFRS Family Members in Grape

In this study, the protein sequences of FRS gene family in Arabidopsis were compared with the grape genome through BLASTP, resulting in the identification of 46 genes exhibiting high homology. Meanwhile, a total of 51 genes satisfying specific criteria were screened from the grape genome based on characteristic domains (PF03101, PF04434 and PF10551) associated with the FRS gene family. The overlapping genes identified by both methods were synthesized using Venn diagram in TBtools v2.210, while their protein sequences were individually verified using SMART and CDD in NCBI databases. Ultimately, a set of 43 grape *VvFRS* family genes was successfully identified.

We designated the grape *VvFRS* family members from *VvFRS1* to *VvFRS43* based on the sequential gene positions on chromosomes indicated by their Locus IDs (Table 1). The physiological and biochemical characteristics of these *VvFRS* genes were investigated, including their protein length, molecular weight, protein isoelectric point (pI), instability index, aliphatic index, hydrophilicity, and subcellular localization. The average length of amino acid sequences for all VvFRS was 602 aa, with a range from 209 aa (VvFRS23) to 985 aa (VvFRS1). Their molecular weights varied from 24.05 kDa to 113.74 kDa. VvFRS15 and VvFRS16 exhibited the lowest (5.23) and highest (9.24) isoelectric point, respectively. In contrast to tomatoes, the FRS family proteins in grape were predominantly slightly acidic [42]. Simultaneously, their instability index ranged from 30.46 (VvFRS42) to 58.27 (VvFRS13), with VvFRS21, VvFRS27, VvFRS37, and VvFRS42 having an instability index below 40, indicating protein stability. The aliphatic index spanned from 63.26 (VvFRS1) to 85.56 (VvFRS3). As a whole, the FRS family proteins in grape demonstrated hydrophilicity, with VvFRS24 exhibiting the highest (−0.923). Finally, it was found that half of the VvFRS proteins were localized within the nucleus.

### 2.2. Phylogenetic Analysis of VvFRSs

**Figure 1:**
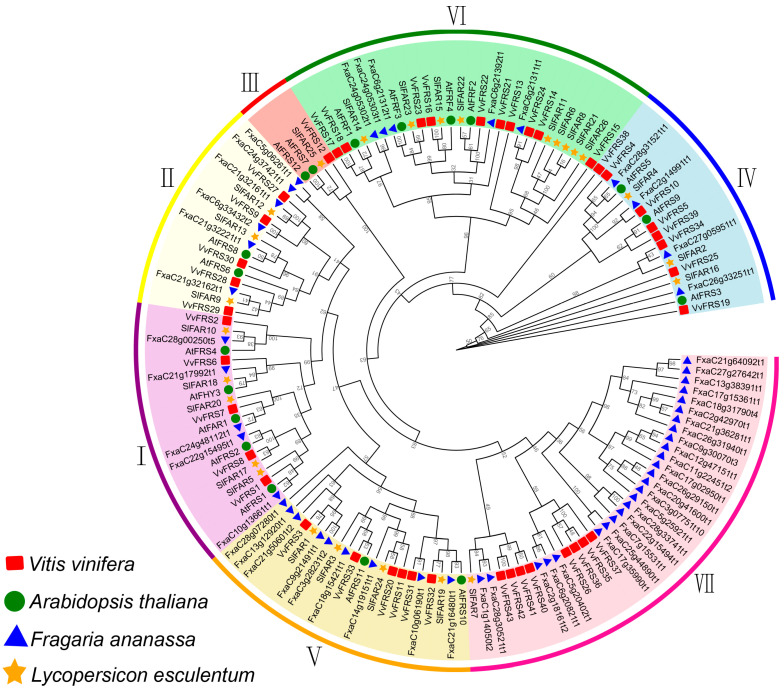
Phylogenetic tree of FRS gene families in different species. Multiple sequence alignments were performed with MEGA 11. Different colors on the genes and the outer circle represented different subsets of FRS genes. The green circle symbolized Arabidopsis, the orange star represented the tomato, the blue triangle denoted the strawberry, and the red square signified the grape.

To investigate the evolutionary relationship between grape and other species, we utilized MEGA 11 to construct a maximum likelihood (ML) phylogenetic tree based on multiple protein sequences alignment from 18 AtFRSs in Arabidopsis [43], 27 SlFRSs from tomato [42], and 43 VvFRSs from grape. Additionally, we incorporated 54 FRS proteins from strawberry, which belong to the same category of berries as grape but have not been reported previously (Figure 1).

We classified the 142 FRSs into seven subgroups, following Arabidopsis’ classification method and sequence similarity [44]. The results shown that subgroup I contained 20 FRSs from four species. Members belonging to this subgroup played a pivotal role within the overall family. A total of 16 FRSs constituted the second subgroup, with five VvFRSs in grape exhibiting a close phylogenetic relationship to Arabidopsis. Subgroup III comprised only four FRSs, with VvFRS12 being the only one belonging to this subgroup in grape. There were 28 FRSs in subgroup VI, and the Arabidopsis members falling under this subgroup were referred to as FRF. Subgroup VII, composed of members from grape and strawberry (except SlFAR7 in tomato), suggested a relatively close relationship between these two species. We hypothesize that subgroup VII may have recently evolved, driven by an abundance of FRS gene copies in grape and strawberry, promoting the emergence of novel protein subtypes.

### 2.3. Synteny Analysis of VvFRSs in Grape

**Figure 2:**
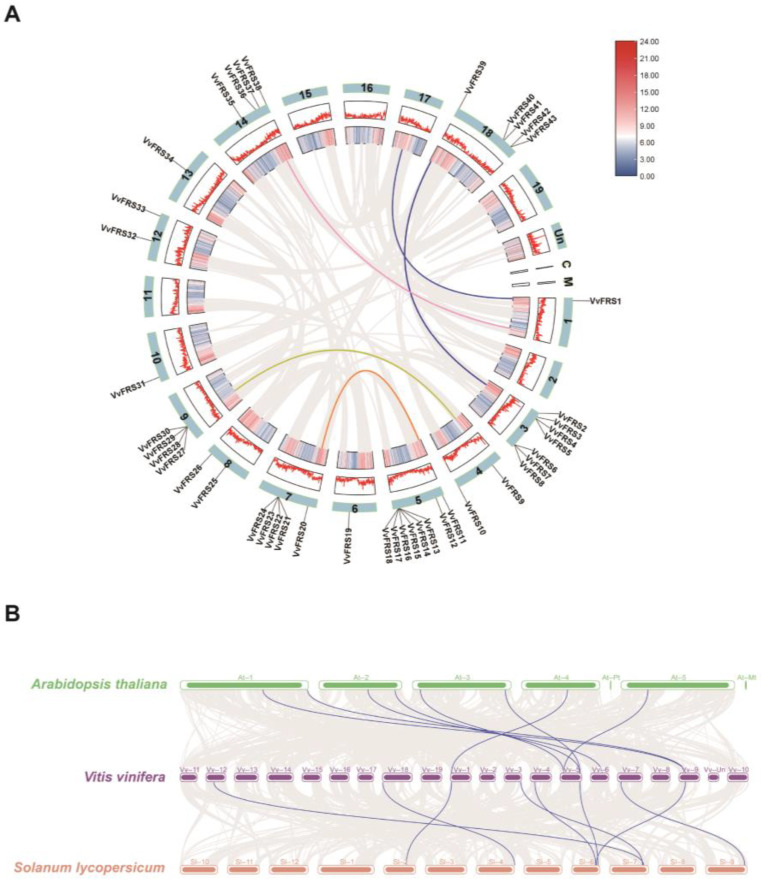
Synteny analysis of *VvFRS* genes. (**A**) Collinearity analysis of *VvFRSs*. Small segments represented different chromosomes. The gray lines represented all collinear blocks in the grape genome, the colored lines represented gene pairs between the *VvFRS* genes or between them and other genes. (**B**) Synteny analysis of FRS genes between Arabidopsis, tomato and grape. The gray lines in the background showed collinearity between the grape and Arabidopsis genomes, the grape and tomato genomes. The blue lines indicate gene pairs of *VvFRSs*.

To further investigate the evolutionary relationships among *VvFRS* genes, we analyzed collinearity maps for the 43 genes located on grape chromosomes (Figure 2A). First of all, the diagram provides insights into the chromosomal distribution of the *VvFRS* genes. It revealed that the *VvFRS* genes were randomly distributed across 13 chromosomes in grape. Chromosomes 3 and 5 carried the highest number of these genes. Most genes in subgroup I were located on chromosome 3, while subgroup II genes were mainly found on chromosome 9. The distribution pattern of gene subgroups IV and V genes appeared relatively scattered as they were localized on five different chromosomes. In general, genes within the same subgroup were generally clustered on the same chromosome.

A total of five collinearity relationships were identified. The pink and blue lines in the figure indicate relationships between *VvFRS* and other family genes, though these are not the focus of this analysis. Notably, three gene pairs within *VvFRS* displayed collinearity: *VvFRS4*/*VvFRS39*, *VvFRS9*/*VvFRS27*, and *VvFRS11*/*VvFRS20*. Studies have reported that the main causes of gene family expansion include genome polyploidy, tandem duplication, segmental duplication, exon duplication, and reorganization [45]. After conducting MCScanX analysis, our study revealed that segmental duplication played a pivotal role in driving the evolution of *VvFRS* genes in grape.

We also identified collinear relationships among the FRS family in Arabidopsis, tomato, and grape. Figure 2B illustrates the presence of eight pairs of homologous genes between Arabidopsis and grape, as well as eight pairs between tomato and grape. The Ka/Ks ratios of the gene pairs between grape and the aforementioned two species were also determined simultaneously (Table S1). The ratios were all below 1, indicating that purifying selection had occurred during the process of evolution.

### 2.4. Conserved Motifs of Grape VvFRS Members

**Figure 3:**
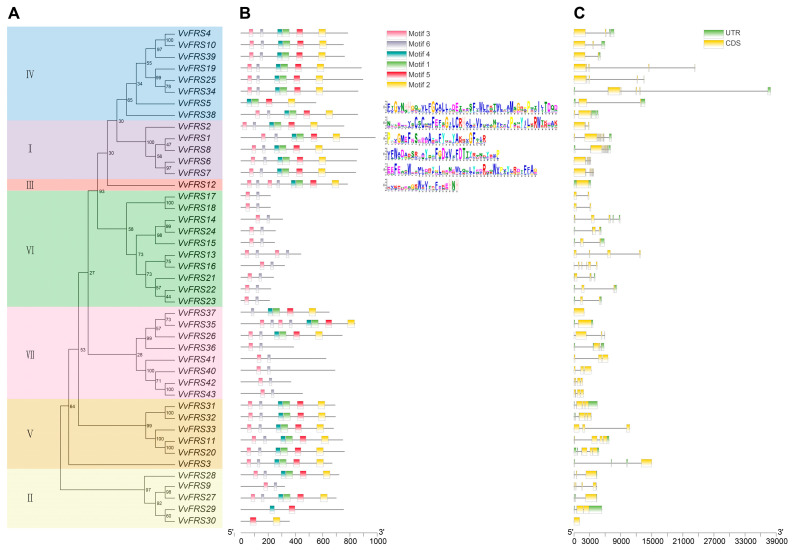
Analysis of the conserved motifs of VvFRS proteins. (**A**) Evolutionary tree of the *VvFRS* gene family. (**B**) Conserved motifs of VvFRS proteins. The top six motifs identified from the grape proteins obtained by the MEME analysis. Different motifs and their positions in each VvFRS member were represented by differently colored boxes. The order of motifs corresponds to their position within individual protein sequences. The sequence logos of conserved motifs were listed at the right. (**C**) Structural analysis of *VvFRS* genes. Green rectangles, yellow rectangles, and black lines represent UTR, CDS, and introns, respectively. Scale bar at bottom estimates lengths of protein and gene sequences.

Six conserved motifs were identified, arranged sequentially from the N-terminal to the C-terminal: motif 3, motif 6, motif 4, motif 1, motif 5, and motif 2 (Figure 3). It was evident that FRS proteins belonging to the same subfamily in the evolutionary tree predominantly possessed similar or identical motifs. VvFRS12, which is homologous to subgroup III in Arabidopsis, contained two copies each of motif 3 and motif 6. These duplications were presumed to form two repeating N-terminal DNA binding domains known as WRKY-GCM1 zinc-finger DNA-binding domain (PF03101). Additionally, most members within grape subgroup VI only possessed motif 3 and motif 6. In Arabidopsis, their closest relatives were represented by four AtFRF which also solely consist of DNA-binding regions within their protein sequences. This indicated functional distinctions among different subsets of grape VvFRS protein members. Similarly, we have also summarized the regional distribution of the coding sequence (CDS) and untranslated regions (UTR) for the *VvFRS* family genes. This analysis reveals that genes within the same subgroup exhibit relatively consistent intron–exon structures.

### 2.5. Cis-Elements in the Promoters of VvFRSs

**Figure 4:**
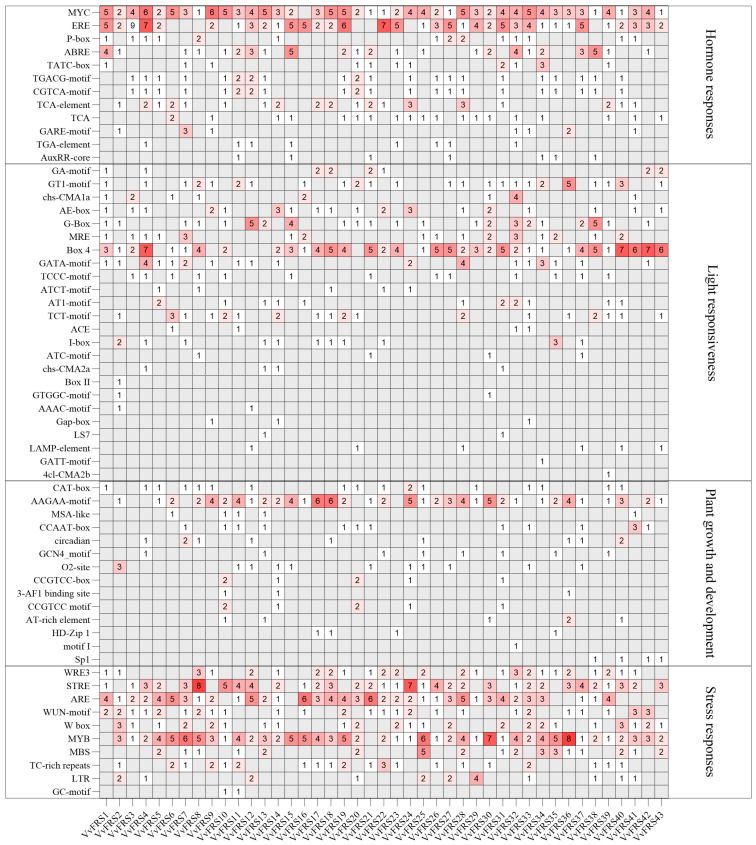
The numbers of cis-element in the promoter of *VvFRSs*.

The cis-acting elements refer to specific DNA sequences located upstream of gene coding sequences, which serve as protein-specific binding sites involved in the initiation and regulation of transcription [46]. To investigate the regulatory mechanisms, metabolic networks, and gene functions of *VvFRS* family members, we extracted the putative promoter sequences from the 2000 bp region upstream of each gene and compared them with the PlantCARE database (Figure S1). Four classes of cis-acting elements were identified in 43 *VvFRS* genes [47]. The promoters of the *VvFRS* genes contained cis-elements associated with plant hormones, light response, the growth and development of plants, and stress response (Figure 4). The photoresponsive cis-elements in *VvFRS* promoters were predominantly represented (40% of total cis-element types). Among these elements, Box 4 exhibited widespread distribution. Promoters of *VvFRS4*, *VvFRS40* and *VvFRS42* contained seven instances of Box 4 element binding sites, indicating that the light signaling pathway protein bound with this cis-element had the most significant regulation on the grape FRS family. G-box, which had a 24% distribution of photoresponsive cis-elements in the Maize FRS family [48], was second only to Box 4 in grapes, with an abundance of approximately 10%.

Although stress-related cis-elements were less abundant, they were the most common in all promoters, comprising 32.9% of the total. The MYB cis-element, associated with high salt and low temperature stress, was the most frequent, present in all gene promoters except for *VvFRS1* and *VvFRS21*. There were 10 gene promoters within the *VvFRS* family that contained five or more MYB elements. At the same time, more ARE cis-elements were found in the promoters of each gene, accounting for 19.32% of the total abundance of stress response elements. This finding further indicated that the FRS family genes were also involved in anaerobic induction. Hormone-related cis-elements were second only to stress-related elements in abundance, with 136 MYC elements accounting for 30.3% of all hormone-associated cis-elements. MYC was associated with methyl jasmonate, suggesting that the FRS family in grape may be involved in the response to the jasmonate pathway. The ethylene-related cis-elements ERE and ABA (abscisic acid)-related cis-element ABRE were also widely distributed among most *VvFRS* genes. Similarly to other species, there appeared to be little correlation between the FRS family and growth of plants as observed in this study [31].

### 2.6. Structural Features of VvFRS Proteins

We utilized online prediction tools to generate the secondary structure data for 43 VvFRS proteins (Table 2). The predominant secondary structure elements observed in grape VvFRS proteins included α-helix, extended strand, β-turn, and random coil. Overall, α-helix and random coil constituted the major proportion within this family. Notably, VvFRS28 (subgroup II) exhibited an α-helix conformation spanning 222 amino acids, accounting for 62.54% of its total sequence. In contrast, VvFRS29 had 68.12% of its amino acids in a random coil configuration—the highest proportion in the VvFRS family.

Meanwhile, to gain a more comprehensive understanding of the structural characteristics of each VvFRS protein, we employed the SWISS-MODEL tool for online prediction and constructed 43 protein models. These models were then classified based on their subgroups in the evolutionary tree (Figure S2). It was evident that proteins belonging to the same subgroup exhibited similarities in their three-dimensional structures. Notably, within the first subgroup, five proteins displayed a close relationship with FHY3/FAR1 in Arabidopsis and possessed approximately half of their structure represented by random coils. At the same time, the fourth subgroup of proteins closely related to the four AtFRFs in Arabidopsis exhibited a distinct α-helix structure simultaneously. Consequently, it was reasonable to speculate that proteins within the same subgroup were likely to share similar functions due to their analogous structural features.

### 2.7. Protein Interaction Networks of VvFRS

**Figure 5:**
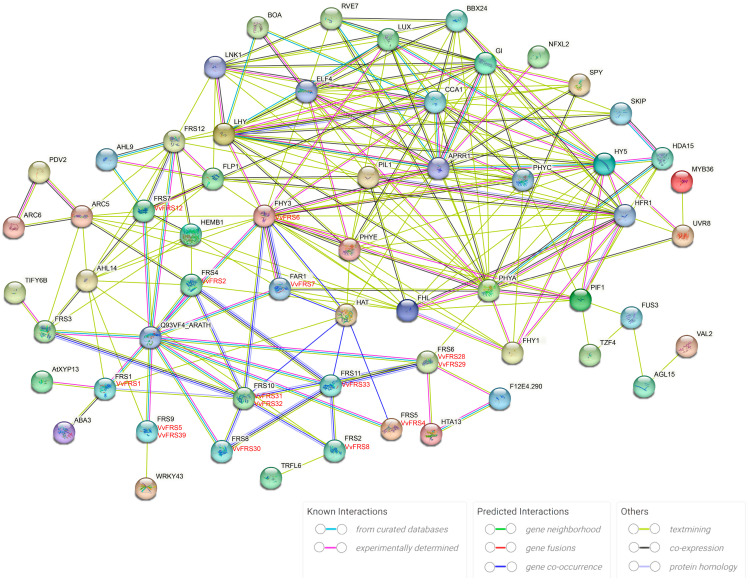
Putative protein interaction network of VvFRSs. The homologous proteins in grape and Arabidopsis were shown in red and black, respectively. The colors of the lines indicated the different types of evidence for the prediction of the protein interactions network.

To investigate the potential interactions and predict the biological functions of VvFRS proteins, we utilized STRING software to integrate VvFRS into an association model of Arabidopsis (Figure 5). It is worth noting that VvFRS6 and VvFRS7 in grapes correspond to AtFHY3 and AtFAR1 in Arabidopsis, respectively. We hypothesized that similar interactions may occur between VvFRS6 and VvFRS7 leading to dimer formation. The relationship between AtFRS4 and VvFRS2 was the closest among all. Previous studies have demonstrated that AtFRS4 can interact with AtFHY3 to enhance ARC5 expression [13], suggesting a potential involvement of VvFRS2 in chloroplast division processes in grape cells. Furthermore, FRS7 and FRS12 were capable of forming repressor protein complexes that partially regulated clock output by controlling the expression of GI (GIGANTEA) and PIF4 [21]. Therefore, it was plausible that VvFRS12 may interact with VvFRS6 and VvFRS7, playing a role in grape flowering and photoperiod regulation. Additionally, VvFRS1 (corresponding to AtFRS1) was potentially implicated in seed storage and ABA signaling pathways. VvFRS30 (corresponding to AtFRS8) and VvFRS33 (corresponding to AtFRS11) may exhibit co-expression [49].

### 2.8. Expression Analysis of VvFRS in Different Tissues

**Figure 6:**
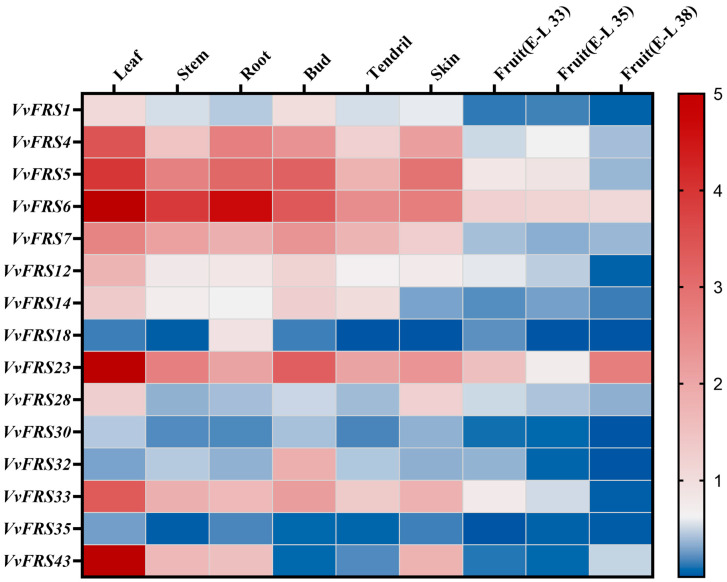
The expression profiles of *VvFRS* in different tissues. Nine developmental tissues were used to analyze expression patterns. These included leaves, stems, roots, buds, and tendrils from the vine, as well as fruits at various stages of development and mature skins from grape berries. Red indicates high levels of transcript abundance, whereas blue indicates low levels. The color scale is shown on the right side.

Exploring the expression patterns of *VvFRS* in grape tissues were of paramount importance for elucidating their potential functions and comprehending their roles in plant physiology. Due to the substantial workload, we prioritized 15 important and representative genes from all *VvFRS* genes for subsequent RT-qPCR experiments (Figure 6). First, subgroup I in Arabidopsis is pivotal within the entire FRS family. Notably, the extensively studied FHY3 and FAR1 correspond to *VvFRS6* and *VvFRS7* in grapes, respectively. Additionally, considering other evolutionary clades within subgroup I, we included *VvFRS1* as another target, which corresponds to *AtFRS1* in Arabidopsis and may be implicated in seed storage and ABA signaling pathways (Figure 5). In subgroup II, *VvFRS28* and *VvFRS30* are homologous to *AtFRS6* and *AtFRS8* in Arabidopsis, respectively, while *AtFRS8* and *AtFRS11* (corresponding to *VvFRS33* in subgroup V) may exhibit co-expression. Subgroup III contains only one gene, *VvFRS12*. In subgroup IV, *VvFRS4* and *VvFRS5* correspond to *AtFRS5* and *AtFRS9* in Arabidopsis, respectively, which play a critical role in the regulatory network of FRS family proteins predicted in this study. Finally, in subgroups VI and VII, several genes with relatively distant evolutionary relationships were selected for further investigation. Furthermore, during our examination of fruit development, we specifically collected and analyzed fruits at E-L-33, E-L-35, and E-L-38 stages according to the refined Eichhorn–Lorenz system for the precise characterization of grape developmental periods [50].

Firstly, we observed a predominant distribution of FRS family genes in vegetative organs such as leaves, stems, roots, buds, and tendrils rather than reproductive organs such as fruits and skins. The expression levels of the FRS family were particularly high in leaves, with *VvFRS1*, *VvFRS6*, and *VvFRS7* from subgroup I and *VvFRS4* and *VvFRS5* from subgroup IV accounting for 37.64% of the total expression level of the selected FRS genes in leaves. These genes were also highly expressed in stems, roots, and buds, indicating that closely related FRS genes may exhibit similar tissue-specific expression patterns and functional roles. From an individual gene perspective, certain members within this family displayed preferential expression in specific tissues; for instance, *VvFRS43* exhibited specific expression solely in grape leaves. *VvFRS6* demonstrated the highest expression among stems, roots, buds and tendrils. However, its expression in roots was reduced by 74.08% compared to that in leaves, suggesting that *VvFRS6* may primarily function in grape leaves. We hypothesized that similar to *AtFHY3* in Arabidopsis, *VvFRS6* was involved in light signal transduction and chlorophyll synthesis processes in grapes. The expressions of FRS family genes in grape fruit and skin were relatively low, with *VvFRS4* and *VvFRS5* in subgroup I and *VvFRS6* in subgroup IV exhibiting higher expression levels in grape skin. In the fruit, most of the FRS genes were detected at significantly low levels. When considering the comparison of fruits at different developmental stages, it can be observed that the expression of most *FRS* genes remained relatively stable throughout fruit development, which may be attributed to their consistently low expression levels.

At the same time, we aimed to investigate whether the expression patterns of *VvFRS* family members in different grape tissues differed from those in Arabidopsis. Therefore, we individually searched the databases for grape and Arabidopsis to explore the expression profiles of FRS family genes in leaves, stems, roots, flowers, and seeds during their respective growth stages. The results were organized based on the evolutionary tree classification of grape *VvFRS* presented in this study. Following the data processing method in the previous study, the absolute value of gene expression in a tissue was divided by the average absolute value of all gene expressions in that tissue [51]. Different colors were used to represent these levels (Figure S3). Our findings aligned with the information retrieved from the database. Amongst FRS family members in both species, subgroups I and IV exhibited significantly higher gene expression levels compared to other subgroups. Notably expressed in vegetative organs and reproductive organs were three closely related genes: *AtFHY3*, *VvFRS6*, and *VvFRS7*. Additionally expressed in seeds and roots were *VvFRS5* and *VvFRS10*, whose closest relatives is *AtFRS9* in subgroup IV. Furthermore, there existed a distinct gene belonging to subgroup VI called *VvFRS23* that has not been found to exhibit substantial tissue-specific expression but consistently displayed high expression levels as observed throughout our experiments.

### 2.9. Expression Analysis of VvFRS Under Different Treatments

**Figure 7:**
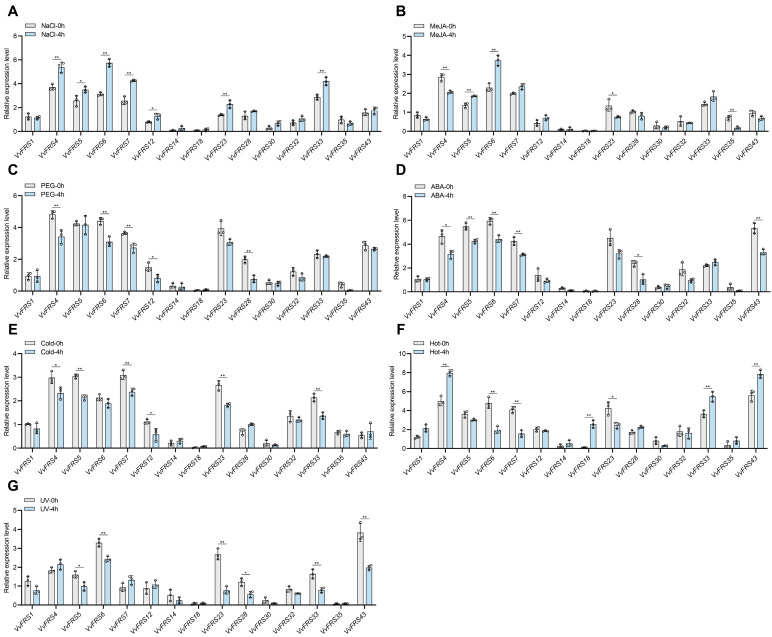
The expression profiles of *VvFRS* in different abiotic stress. Gene expression patterns of 15 *VvFRS* genes in response to (**A**) NaCl, (**B**) methyl jasmonate (MeJA), (**C**) drought, (**D**) abscisic acid (ABA), (**E**) cold, (**F**) hot, and (**G**) ultraviolet (UV) stresses. The samples of the wild-type ‘Thompson Seedless’ hydroponic seedlings were collected at 0 h and 4 h post-treatment. *VvActin* was used as a control. All data are means ± SD; n = 3 biological replicates. Asterisks indicate significant differences compared to the control (* *p* < 0.05, ** *p* < 0.01, two-tailed Student’s *t*-test).

The growth of plants is intricately linked to the environment, and abiotic stress plays a pivotal role in constraining the growth of cash crops. Analyzing the involvement of FRS family genes in diverse abiotic stress processes within the grape holds immense significance for enhancing agricultural productivity and augmenting the economic traits of grape products. Consequently, we used RT-qPCR to examine representative FRS genes in tissue culture seedlings of ‘Thompson Seedless’ subjected to various abiotic stresses. The plants were treated to seven abiotic stresses, including salt, drought, high temperature, cold, ultraviolet radiation (UV), ABA, and jasmonic acid (Figure 7).

We found that genes from subgroups I (*VvFRS6*, *VvFRS7*) and IV (*VvFRS4*, *VvFRS5*) exhibited a more pronounced response to various abiotic stresses. An up-regulation trend of *VvFRS* genes was observed in almost all subgroups following salt treatment. Specifically, *VvFRS* members in subgroups I and IV exhibited significant up-regulation, with a notable increase of 65.8% observed in *VvFRS6* expression. Furthermore, although most *VvFRS* genes did not exhibit a noticeable response process after jasmonic acid treatment in grape tissue culture seedlings, it was noteworthy that the *VvFRS6* played a more prominent role. Specifically, 33.46% of the expression of *VvFRS6* was up-regulated by jasmonic acid treatment, suggesting its potential positive regulatory role in the jasmonic acid signaling pathway. ABA and cold treatments down-regulated the expression levels of many *VvFRS* genes. This discrepancy may be attributed to different species having varying response times to abiotic stresses such as ABA, drought, and cold or that exceeding the rapid response time could lead to the down-regulation of gene expression. Interestingly, the grape tissue culture seedlings exhibited minimal responsiveness to high temperature stress as evidenced by unaltered expression levels of most *VvFRS* family genes.

## 3. Discussion

The FRS gene family consisted of transcription factors derived from MULE transposase, which played a pivotal role in plant light signal response, growth response, stress response, and other essential life processes by regulating the expression of downstream genes [24]. In Arabidopsis, a total of 14 *FRS*/*FRF* genes have been identified and extensively studied for their functions and properties [10,49]. Moreover, the presence of FRS families has also been reported in tomato, barley, corn, cotton, eucalyptus, potato cucumber and other plant species. Grape is an economically significant crop widely cultivated in China. However, no comprehensive report on the identification and analysis of the complete *VvFRS* gene family in grape or other berry fruit trees have yet been published. Therefore, we conducted this study to identify 43 members of the *VvFRS* family in grapes for the first time. We analyzed their physicochemical properties and evolutionary relationships while predicting their potential involvement in biological pathways.

### 3.1. The Structure, Evolutionary Characteristics, and Functions of VvFRSs

In this study, the 43 *VvFRS* genes of grapes were found to be randomly distributed across its 13 chromosomes, indicating their distinct expression patterns. Physicochemical analysis revealed that most VvFRS proteins in grapes exhibited instability, except for VvFRS21, VvFRS27, VvFRS37 and VvFRS42, which was consistent with findings in Arabidopsis [9]. Subcellular localization prediction results suggested that some members of the VvFRS family may localize to cellular components other than the nucleus (Table 1). It is known that transcription factors primarily function intracellularly within cells. Previous studies have shown that AtFRS1, AtFRS8 and AtFRS9 in Arabidopsis lack putative nuclear localization sequences (NLS), but they may utilize atypical NLS for nuclear import or interact with other FRS proteins containing MLS motifs to form protein complexes that translocate into the nucleus and regulate gene expression [10]. Therefore, it is possible that non-nuclear localized VvFRS proteins in grapes can also exert their functions by interacting with other members to form complexes. Additionally, replication or recombination events have occurred among members of the FRS gene family during grape evolution. The collinear analysis revealed the presence of segmental duplications within the *VvFRS* family, while the Ka/Ks ratio indicated that these genes have undergone negative or purifying selection during evolution, which was similar to the results in potato [31]. The presence of segmental duplications provided a basis for evolutionary diversification within the FRS transcription factor families to some extent [52].

We classified the FRS family in Arabidopsis, tomato, strawberry, and grape into seven subgroups based on multiple sequence alignment and phylogenetic analysis (Figure 1). AtFHY3 and AtFAR1 can be found in subgroup I, which play a pivotal role in the entire FRS family, with five genes belonging to this subgroup in grapes. In Arabidopsis, this subgroup is the largest within the FRS family, with homodimers or heterodimers of FHY3 and FAR1 binding directly to promoters to activate transcription of FHY1 and FHL, which regulate phyA translocation into the nucleus under far-red light. This process indirectly influences phyA nuclear import mediated by FHY3 and FAR1 [8]. VvFRS6 was identified as the closest relative of AtFHY3, providing a reference for investigating the potential function of VvFRS6 in grapes.

The apparent expansion of the FRS gene family in subgroup VII of the phylogenetic tree was observed in grapes and strawberries. This may reflect adaptive selection in these two species in response to specific environmental stresses or physiological functions during evolution. First, the FRS gene family plays a critical role in regulating plant responses to abiotic stress [49]. As perennial fruit crops, strawberries and grapes frequently encounter diverse environmental pressures. Gene family expansion could enhance the robustness of stress response networks through functional redundancy or diversification. Additionally, fruit development in strawberries and grapes involves intricate hormonal regulation and the accumulation of secondary metabolites. Members of the FRS family may finely regulate the expression of genes associated with fruit ripening. For instance, the functional differentiation of the *CYP75* gene family in flavonoid synthesis within grapes indicated that gene family expansion might facilitate the optimization of species-specific metabolic pathways, thereby enhancing fruit quality or conferring disease resistance [53]. Furthermore, gene family expansion in grapes and strawberries is often accompanied by tandem duplication or segmental replication events. This replication pattern may accelerate functional innovation. For instance, the expansion of the *GASA* gene family in grapes maintains adaptive mutations via negative selection pressure [54].

Additionally, the differential expression of the WRKY gene family in strawberries under continuous cropping stress implies that expanded genes may play a role in environmental adaptation [55]. The expansion of subgroup VII may enable gene members to differentiate into tissue-specific or stress-specific regulatory modules. Finally, cultivated grapes and strawberries have undergone artificial selection to improve fruit traits, including size, color, and flavor, during domestication. The expansion of the FRS gene family may facilitate fruit phenotypic diversification by regulating downstream target genes, such as anthocyanin synthase genes [56,57]. For instance, alterations in SPL gene family expression during strawberry fruit development and the expansion of terpenoid metabolism-related genes in domesticated grape varieties indicate that gene family dynamics are closely linked to human-imposed selection pressures [58,59]. Overall, the expansion of subgroup VII of the *FRS* gene families in grapes and strawberries likely facilitates their adaptation to ecological niches and domestication requirements by increasing the complexity of stress response networks, refining the regulation of fruit development, and enhancing genomic plasticity.

The conserved domain analysis of grape FRS genes also aligned with their developmental and evolutionary classification. The sequences of FRS family members showed homology and conservation across land plants. Most VvFRS members in subgroup VI possessed only motif 3 and motif 6 (Figure 3). In Arabidopsis, their closest relatives were four AtFRF genes that also exclusively contain DNA-binding regions within their protein sequences. This suggested functional distinctions among different subgroups of grape VvFRS proteins. The protein-level conservation of VvFRS was confirmed through phylogenetic and conserved domain analyses, suggesting that genes within the same subbranch likely share similar functional roles.

### 3.2. The Promoters of VvFRSs Contain Abundant Stress Response Elements

Analysis of cis-elements in the promoter region of *VvFRSs* revealed an abundance of abiotic stress response and hormonal elements (Figure 4). Notably, MYB cis-elements associated with high salt and low temperature conditions were particularly prevalent, which aligning with findings in Eucalyptus [28]. While ARE cis-elements were also found across all gene promoters, suggesting the involvement of FRS family in abiotic stress processes such as salt, low temperature, and anaerobic induction. Additionally, MYC elements were present in almost all *VvFRS* gene promoters. The presence of MYC suggested the potential participation of the grapevine FRS family in the jasmonate pathway response mediated by methyl jasmonate. As ABA synthesis can be induced by various stresses, ABA was considered a plant stress hormone [60]. Many genes involved in ABA signaling contained ABRE elements within their promoter regions; these genes often play crucial roles in plants’ response to abiotic stress [61].

### 3.3. VvFRS Proteins Interaction Network

We conducted an analysis on the secondary structure of 43 VvFRS proteins and observed a predominant presence of α-helices and random coils (Table 2). The prevalence of coiled helical domains in most FRS proteins suggested their potential for forming homologous and/or heterodimers, as well as interacting with various partners to regulate a wide range of signal transduction processes. In fact, previous reports have provided evidence for the homologous dimerization of FAR1 and FHY3 in Arabidopsis, along with their interactions with other proteins [9,62].

Based on the interaction network of Arabidopsis FRS proteins, we have predicted and analyzed the potential involvement of VvFRS proteins in various biological developmental processes. In Arabidopsis, FHY3 and FAR1 interact to form dimers or heterodimers, regulating the transcription of various target genes, with FHY3 playing the central role [9]. Additionally, CCA1, LHY, and PIF1 in Arabidopsis interacted with FHY3, resulting the in inhibition of *ELF4* and *HEMB1* transcriptional activity [15,19]. Simultaneously, HY5 also interacted with FHY3 promoting the expression of *ELF4* and *COP1* [11,19]. Furthermore, EIN3-FHY3 interaction enhanced *PHR1* expression [50]. These clues collectively suggested that VvFRS6 and VvFRS7 might play crucial roles in grape photomorphogenesis, seed germination, flowering time, chlorophyll synthesis and other processes (Figure 5). Furthermore, VvFRS2 in grape corresponded to the AtFRS4 in Arabidopsis. Considering that the interaction between AtFRS4 and AtFHY3 enhanced ARC5 expression [13], it suggested that VvFRS2 might be involved in chloroplast division in grape. Additionally, FRS7 and FRS12 can form inhibitory protein complexes that partially regulated clock output. Additionally, it has been identified that FRS7, HON4 (Histone-like protein 4), AHL9 (AT-Hook motif nuclear localized protein 9) and AHL14 can interact with FRS12 in Arabidopsis through tandem affinity purification-mass spectrometry (TAP-MS) [21]. Therefore, there was potential for an interaction between VvFRS12 with VvFRS6 and VvFRS7 to contribute to grape flowering regulation under photoperiodic conditions.

### 3.4. VvFRF6 Plays an Important Role in Stress Resistance

Exploring the expression patterns of FRS family genes in grape tissues holds significant importance for comprehending their functionalities. Hence, we investigated the expression profiles of representative genes in different grape subgroups across distinct organs. Generally, *VvFRS* genes were predominantly distributed in leaves, with a higher number of expressed genes observed in subgroup I and subgroup IV. Notably, the expression level of *VvFRS6* was significantly elevated across all tissues compared to other *VvFRS* members (Figure 6). Additionally, we examined the response of *VvFRS* genes to abiotic stress (Figure 7). Subgroups I and IV exhibited substantial up-regulation after exposure to salt stress, with a remarkable increase observed in *VvFRS6* expression. This aligns with previous studies on tea plants, where genes in similar subgroups (*CsFRS-5* and *CsFRS-12* from subgroup I, and *CsFRS-22* and *CsFRS-24* from subgroup IV) showed high up-regulation under stress conditions such as drought and salt treatment [29]. The functional similarities between these genes suggest that closely related *VvFRS* genes might share common regulatory roles in stress responses. The FRS gene might function as a transcription factor during the initial stages of grape’s response to salt stress, but they may become inactive once the plant has completed the process of excreting and injecting of hydrogen and sodium ions. This is consistent with previous reports that FAR family genes in Eucalyptus grandis also responded early to salt stress, with FAR1/FHY3 expression peaking at 6 h and declining at 12 h [28]. High temperature stress had little effect on *VvFRS* gene expression in grape, similar to findings in potato where StFRS expression remained unchanged under heat stress [31].

We hypothesized that the *VvFRS* family may play a role in regulating abiotic stress responses via diverse molecular mechanisms. First, the promoter regions of *VvFRS* genes are enriched with plant hormone elements (e.g., ABA, MeJA, and ethylene) as well as abiotic stress elements (e.g., low temperature and salt stress) (Figure 4). These elements may recruit specific transcription factors (TFs) to activate *VvFRS* gene expression in response to external stress signals [31]. Meanwhile, the expression analysis revealed that the *VvFRS* gene exhibits spatio-temporal specific expression patterns across different grape tissues and developmental stages (Figure 6 and Figure 7). For instance, the expression of certain *VvFRS* members were significantly upregulated during fruit ripening or under salt stress conditions. This upregulation may enhance cellular tolerance to oxidative damage by modulating the synthesis or accumulation of secondary metabolites, such as flavonoids and antioxidants [63]. Furthermore, VvFRS may interact with other regulatory proteins, including MYC and MYB transcription factors, to form synergistic or antagonistic networks. These networks could integrate hormone signaling pathways, such as ABA and MeJA, thereby enhancing stress resistance [64,65].

## 4. Materials and Methods

### 4.1. Plant Materials and Treatments

The experimental vineyard was located at the Shangzhuang Experimental Station of China Agricultural University, Beijing, China (116.11° E, 40.08° N, temperate continental monsoon climate), and samples were collected during the 2024 vintage. The vines were planted in a trellis system with single cordons. The spacing within and between the vines rows was 1.5 m and 3.0 m, respectively. The different grape organ tissues (leaves, stems, roots, buds, tendrils, fruits and skins) were collected from soil-grown ‘Thompson Seedless’ (*Vitis vinifera* L.) vines. As for tissue culture seedlings used for stress treatment, the young stem segments at the apex of the grape branches were excised and transported to the laboratory. After being disinfected with alcohol and sodium hypochlorite, they were placed onto a rooting medium and cultivated at 25 °C under a photoperiod of 16 h light/8 h dark. The sterile tissue culture seedlings with between four and five juvenile leaves were obtained after approximately 2 months of growth. The uniform and vigorous seedlings were carefully selected, removed from solid medium, and subsequently cultivated hydroponically in Murashige and Skoog (MS, Phyto Tech, Overland Park, KS, USA) liquid medium.

### 4.2. Identification of VvFRS Family Genes in Grape

To identify *VvFRSs* in grape, the FAR1/FHY3 DNA binding domain (PF03101), SWIM domain (PF04434), and MULE transposase domain (PF10551) were extracted from the Pfam database (http://pfam.xfam.org/, accessed on 11 July 2024) [66]. The FRS genes in Arabidopsis were compared with the grape genome using the blast method simultaneously. The *VvFRS* protein sequences were verified in the NCBI Conserved Domains Database (http://www.ncbi.nlm.nih.gov/Structure/cdd/wrpsb.cgi, accessed on 13 July 2024) and on the SMART website (http://smart.embl-heidelberg.de, accessed on 13 July 2024) [67]. Proteins lacking the FRS conserved domains were removed. The protein sequences of *VvFRS* family members were uploaded to the Expasy website (https://web.expasy.org, accessed on 20 July 2024), and their molecular weight, isoelectric point, and other physicochemical properties were predicted. Cell-PLoc 2.0 was used for predicting subcellular localization (Plant-mPLoc server (sjtu.edu.cn, accessed on 1 August 2024)). After downloading the GFF3 file of the grape genome, TBtools v2.210 software was utilized to perform chromosome location map for *VvFRS* genes.

### 4.3. Phylogenetic Evolutionary Analysis of FRS Genes in Grape

The FRS protein sequences of Arabidopsis, tomato and strawberry were downloaded from the online database Ensembl (https://asia.ensembl.org/index.html, accessed on 15 August 2024). The MEGA 11 software was used to construct a phylogenetic tree of FRS protein sequences by applying the Maximum Likelihood method [68]. The bootstrap replication value was set as 1000. The Evolview (https://www.evolgenius.info//evolview-v2, accessed on 20 August 2024) website was used to improve the appearance of the evolutionary tree.

### 4.4. Collinearity Analysis of Grape VvFRS

The genome files and genomic annotation file of the related FRS gene families in grape, Arabidopsis and tomato were downloaded, and the collinearity analysis was performed by using the ‘Text Merge for MCScanX’ function in TBtools v2.210 software. The Multiple Covariance Scanning Toolkit (MCScanX, default parameters) was used to analyze the duplication events. Finally, the results were visualized by ‘Multiple Systeny Plot’ function in TBtools v2.210 [69]. The Ka/Ks of all tandemly duplicated gene pairs were calculated using KaKs_Calculator [70].

### 4.5. Gene Structure and Conserved Motifs Analysis of VvFRS

The distribution of the conserved motifs based on amino acid sequence was conducted with the online MEME website (http://meme-suite.org/tools/meme, accessed on 25 August 2024) [71]. The corresponding motif information and the evolutionary tree information of grape FRS family derived from MEGA 11 were combined to be analyzed via the ‘gene structure view’ function of TBtools to visualize the conserved motifs of grape FRS members.

### 4.6. Analysis of Cis-Elements in Promoters

The 2000 bp DNA sequences in the grape FRS gene upstream regions were screened as promoter sequences using TBtools v2.210 software. The PlantCARE database (https://bioinformatics.psb.ugent.be/webtools/plantcare/html/, accessed on 6 September 2024) was used to search for cis-regulatory elements in grape gene promoter regions [46].

### 4.7. Structural Characteristics and Interaction Network of VvFRS Proteins

The NPS online website (https://npsa.lyon.inserm.fr/cgi-bin/npsa_automat.pl?page=/NPSA/npsa_sopma.html, accessed on 13 September 2024) was utilized for predicting the secondary structure of the proteins. The SWISS-MODEL tool (https://swissmodel.expasy.org/, accessed on 14 September 2024) was employed to generate tertiary structure models of VvFRS proteins. The color gradient in the models from blue to red indicated the sequential transition from the N-terminal to the C-terminal region of the proteins. For the analysis of protein interaction network, STRING online prediction tool (https://cn.string-db.org/, accessed on 24 September 2024) was used [72].

### 4.8. Quantitative Real-Time PCR Analysis

To induce various stress conditions, the roots of selected plants were submerged in MS medium containing 100 mM NaCl (Sangon Biotech, Shanghai, China) or 15% PEG6000 (Macklin, Shanghai, China) to simulate salt stress and drought stress. Tissue culture seedlings were subjected to artificial climate chamber maintained at 4 °C or 40 °C to simulate cold stress and high temperature stress. Additionally, leaves of seedlings were sprayed with a solution containing 100 μM ABA (Solarbio, Beijing, China) or 50 μM jasmonic acid (Solarbio, Beijing, China) until dripping occurred. Finally, the grape seedlings were exposed to ultraviolet lamps for 8 min to simulate ultraviolet stress. Hydroponic plants under natural conditions were served as controls. Leaves from the same part of three separate plants were collected rapidly and frozen in liquid nitrogen before being stored at −80 °C.

The HiPure HP Plant RNA Mini Kit (Magena, Beijing, China) was employed for the extraction of total plant RNA from various tissues and organs of ‘Thompson Seedless’, as well as samples collected under diverse stress environments and hormone conditions. The RNA concentration was determined using a NanoDrop ND 1000 spectrophotometer (Thermo Fisher Scientific, Waltham, MA, USA), and the integrity of the RNA samples were assessed through agarose-gel electrophoresis. The first cDNA synthesis was performed with 1 μg total RNA using HiScript II Q RT SuperMIX for qPCR (Vazyme, Nanjing, China). ChamQ SYBR qPCR Master Mix (Vazyme, Nanjing, China) was utilized for RT-qPCR on an IQ5 Real-Time PCR System (Bio-Rad, Hercules, CA, USA). All primers used for RT-qPCR analysis were designed via Primer-Blast (https://www.ncbi.nlm.nih.gov/tools/primer-blast/, accessed on 8 October 2024) and listed in Table S2. Three independent biological replicates and technical replicates were analyzed for each sample. The relative expression levels were calculated following the 2^−∆∆Ct^ method [73].

### 4.9. Statistical Analysis

The data were expressed as the mean and standard deviation (SD) of three biological and technical replicates. Asterisks indicate significant differences compared to the control (* *p* < 0.05, ** *p* < 0.01, two-tailed Student’s *t*-test). All statistical analyses were conducted using Excel (Microsoft Corp., Redmond, WA, USA) and GraphPad Prism software (version 9.0).

## 5. Conclusions

This study conducted a comprehensive and systematic analysis of the FRS family in grape. A total of 43 *VvFRS* genes were identified and categorized into seven subgroups. Their physical and chemical properties were predicted, their phylogenies and gene structures were analyzed, and their replication events and collinearity with other species were examined. Furthermore, their protein structures and interactions with other proteins were explored, while their promoter cis-elements mainly participated in abiotic stress response and hormone treatment. The quantitative analysis revealed the expression pattern of *VvFRS* genes in grape tissues and abiotic stress conditions. It was observed that these genes predominantly expressed in leaves, with a significant increase in *VvFRS6* expression following salt stress exposure in grapes. Although these experiments have partially addressed the gap in systematic research on *FRS* family genes in grapes, this study lacks direct physiological function validation of *VvFRS* family members. Future investigations could focus on conducting a series of experiments to explore the salt tolerance of VvFRS6. Overall, these outcomes will serve as a foundation for further investigations into the *VvFRS* family while contributing to an enhanced understanding of the mechanisms underlying VvFRS-mediated stress responses in grapes.

## Figures and Tables

**Table 1:** Characterization of FAR1-RELATED SEQUENCE (FRS) genes in grape.

Gene ID	Locus ID	Protein Length (aa)	Molecular Weight (Da)	Theoretical Isoelectric Point	Instability Index	Aliphatic Index	Grand Average of Hydropathicity	Subcellular Localization
*VvFRS1*	Vitvi01g00116_t001	985	113,743.53	8.52	56.77	63.26	−0.694	Nucleus
*VvFRS2*	Vitvi03g04004_t001	755	87,847.54	6.68	52.81	72.23	−0.583	Nucleus
*VvFRS3*	Vitvi03g00193_t001	752	85,954.31	8.69	40.56	85.56	−0.283	Chloroplast
*VvFRS4*	Vitvi03g01475_t001	783	89,339.54	6.16	47.37	70.59	−0.528	Chloroplast
*VvFRS5*	Vitvi03g00266_t001	549	62,553.56	6.88	40.42	69.87	−0.512	Nucleus
*VvFRS6*	Vitvi03g01239_t001	847	97,096.12	6.20	52.23	66.46	−0.662	Nucleus
*VvFRS7*	Vitvi03g01243_t001	841	96,465.3	8.03	50.23	69.1	−0.571	Chloroplast
*VvFRS8*	Vitvi03g01244_t001	857	98,181.2	6.24	47.67	72.67	−0.54	Chloroplast
*VvFRS9*	Vitvi04g00504_t001	668	77,635.63	6.45	42.17	77.14	−0.438	Chloroplast
*VvFRS10*	Vitvi04g01750_t001	751	85,598.73	6.41	45.31	73.87	−0.45	Chloroplast
*VvFRS11*	Vitvi05g00081_t001	746	85,345.3	6.19	49.93	80.64	−0.288	Nucleus
*VvFRS12*	Vitvi05g01873_t001	781	89,714.38	6.88	43.13	74.55	−0.507	Chloroplast
*VvFRS13*	Vitvi05g01497_t001	439	51,066.38	6.51	58.27	69.75	−0.868	Nucleus
*VvFRS14*	Vitvi05g01498_t001	305	35,433.14	6.65	52.65	78	−0.666	Nucleus
*VvFRS15*	Vitvi05g01499_t001	246	28,349.96	5.23	50.88	71.71	−0.694	Chloroplast
*VvFRS16*	Vitvi05g04445_t001	319	36,844.6	9.24	40.6	83.39	−0.464	Chloroplast
*VvFRS17*	Vitvi05g01506_t001	215	25,134.58	7.06	45.71	68.56	−0.771	Chloroplast
*VvFRS18*	Vitvi05g01510_t001	215	25,082.41	6.34	49.63	67.63	−0.8	Cytoplasm
*VvFRS19*	Vitvi06g01048_t001	883	99,376.01	5.89	48.17	71.7	−0.468	Chloroplast
*VvFRS20*	Vitvi07g00384_t001	758	86,535.53	6.46	49.54	76.15	−0.346	Nucleus
*VvFRS21*	Vitvi07g04446_t001	239	27,624.01	5.27	39.56	70.5	−0.878	Nucleus
*VvFRS22*	Vitvi07g01198_t001	218	25,372.89	8.94	51.09	70.6	−0.843	Nucleus
*VvFRS23*	Vitvi07g01199_t001	209	24,052.29	8.56	57.83	68.42	−0.722	Nucleus
*VvFRS24*	Vitvi07g02480_t001	252	29,153.62	6.32	48.73	68.81	−0.923	Nucleus
*VvFRS25*	Vitvi08g00891_t001	894	100,926.01	6.18	41.1	71.88	−0.49	Chloroplast
*VvFRS26*	Vitvi08g01733_t001	742	84,686.41	5.27	46.39	74.77	−0.431	Nucleus
*VvFRS27*	Vitvi09g00649_t001	718	82,911.95	5.85	37.53	76.14	−0.431	Chloroplast
*VvFRS28*	Vitvi09g04199_t001	355	41,623.75	8.63	45.54	85.41	−0.402	Nucleus
*VvFRS29*	Vitvi09g04200_t001	320	36,041.05	9.20	44.62	70.94	−0.55	Chloroplast
*VvFRS30*	Vitvi09g00650_t001	698	81,102.44	5.37	42.64	74	−0.561	Cytoplasm
*VvFRS31*	Vitvi10g00230_t001	690	80,193.97	5.98	45.84	74.17	−0.416	Nucleus
*VvFRS32*	Vitvi12g00713_t001	692	80,089.01	6.21	46.85	79.57	−0.364	Nucleus
*VvFRS33*	Vitvi12g02073_t001	678	77,793.76	8.08	42.55	77.83	−0.354	Nucleus
*VvFRS34*	Vitvi13g01139_t001	858	97,221.73	6.16	48.68	72.52	−0.451	Cell wall
*VvFRS35*	Vitvi14g00948_t001	647	75,285.04	6.73	46.49	76.57	−0.43	Nucleus
*VvFRS36*	Vitvi14g01432_t001	386	43,770.41	5.77	55.88	64.64	−0.698	Nucleus
*VvFRS37*	Vitvi14g01622_t001	835	96,149.99	6.32	39.69	73.62	−0.523	Chloroplast
*VvFRS38*	Vitvi14g02030_t001	855	97,537.38	6.00	49.11	68.78	−0.483	Nucleus
*VvFRS39*	Vitvi18g00039_t001	759	86,033.65	6.14	46.26	71.71	−0.511	Chloroplast
*VvFRS40*	Vitvi18g01884_t001	689	76,873.44	5.34	49.22	75.99	−0.535	Chloroplast
*VvFRS41*	Vitvi18g03049_t001	623	69,497.33	5.73	47.02	69.52	−0.722	Nucleus
*VvFRS42*	Vitvi18g04647_t001	365	40,838.6	6.07	30.46	68.14	−0.646	Nucleus
*VvFRS43*	Vitvi18g03185_t001	451	50,798.04	5.60	42.97	76.32	−0.644	Nucleus

**Table 2:** Two-dimensional structures of VvFRS proteins.

Name	Sequence Length	Amino Acid Number/Proportion			
		Alpha Helix (Hh)	Extended Strand (Ee)	Beta Turn (Tt)	Random Coil (Cc)
VvFRS1	985	339/34.42%	89/9.04%	30/3.05%	527/53.50%
VvFRS2	755	309/40.93%	81/10.73%	26/3.44%	339/44.90%
VvFRS3	752	346/46.01%	102/13.56%	23/3.06%	281/37.37%
VvFRS4	783	322/41.12%	88/11.24%	25/3.19%	348/44.44%
VvFRS5	549	275/50.09%	51/9.29%	14/2.55%	209/38.07%
VvFRS6	847	319/37.66%	85/10.04%	28/3.31%	415/49.00%
VvFRS7	841	328/39.00%	81/9.63%	25/2.97%	407/48.39%
VvFRS8	857	305/35.59%	84/9.80%	23/2.68%	445/51.93%
VvFRS9	668	310/46.41%	83/12.43%	27/4.04%	248/37.13%
VvFRS10	751	325/43.28%	83/11.05%	27/3.60%	316/42.08%
VvFRS11	746	311/41.69%	82/10.99%	24/3.22%	329/44.10%
VvFRS12	781	332/42.51%	113/14.47%	27/3.46%	309/39.56%
VvFRS13	439	140/31.89%	61/13.90%	18/4.10%	220/50.11%
VvFRS14	305	110/36.07%	39/12.79%	8/2.62%	148/48.52%
VvFRS15	246	81/32.93%	28/11.38%	10/4.07%	127/51.63%
VvFRS16	319	113/35.42%	36/11.29%	11/3.45%	159/49.84%
VvFRS17	215	86/40.00%	29/13.49%	11/5.12%	89/41.40%
VvFRS18	215	85/39.53%	30 /13.95%	10/4.65%	90/41.86%
VvFRS19	883	356/40.32%	103/11.66%	30/3.40%	394/44.62%
VvFRS20	758	329/43.40%	80/10.55%	27/3.56%	322/42.48%
VvFRS21	239	87/36.40%	29/12.13%	13/5.44%	110/46.03%
VvFRS22	218	84/38.53%	29/13.30%	10/4.59%	95/43.58%
VvFRS23	209	84/40.19%	31/14.83%	8/3.83%	86/41.15%
VvFRS24	252	82/32.54%	29/11.51%	12/4.76%	129/51.19%
VvFRS25	894	343/38.37%	103/11.52%	26/2.91%	422/47.20%
VvFRS26	742	314/42.32%	83/11.19%	23/3.10%	322/43.40%
VvFRS27	718	311/43.31%	84/11.70%	29/4.04%	294/40.95%
VvFRS28	355	222/62.54%	25/7.04%	8/2.25%	100/28.17%
VvFRS29	320	48/15.00%	40/12.50%	14/4.38%	218/68.12%
VvFRS30	698	325/46.56%	85/12.18%	23/3.30%	265/37.97%
VvFRS31	690	324/46.96%	80/11.59%	27/3.91%	259/37.54%
VvFRS32	692	316/45.66%	80/11.56%	26/3.76%	270/39.02%
VvFRS33	678	315/46.46%	78/11.50%	27/3.98%	258/8.05%
VvFRS34	858	344/40.09%	90/10.49%	21/2.45%	403/46.97%
VvFRS35	647	314/48.53%	79/12.21%	25/3.86%	229/35.39%
VvFRS36	386	114/29.53%	29/7.51%	8/2.07%	235/60.88%
VvFRS37	835	310/37.13%	111/13.29%	30/3.59%	384/45.99%
VvFRS38	855	336/39.30%	94/10.99%	24/2.81%	401/46.90%
VvFRS39	759	333/43.87%	82/10.80%	26/3.43%	318/41.90%
VvFRS40	689	275/39.91%	69/10.01%	32/4.64%	313/45.43%
VvFRS41	623	288/46.23%	64/10.27%	22/3.53%	249/39.97%
VvFRS42	365	131/35.89%	39/10.68%	14/3.84%	181/49.59%
VvFRS43	451	146/32.37%	56/12.42%	19/4.21%	230/51.00%

## Data Availability

All relevant data are included within the paper and its Supplementary Materials.

## Supplementary Materials

The supporting information can be downloaded at: https://www.mdpi.com/article/10.3390/ijms26104675/s1.
