# Supplementary material for: Genome-Wide Identification and Expression Analysis of the FAR1-RELATED SEQUENCE (FRS) Gene Family in Grape (Vitis vinifera L.)
Source: Int J Mol Sci. 2025 May 14;26(10):4675. doi: 10.3390/ijms26104675 (PMC12112247; doi:10.3390/ijms26104675)
Supplement: Supplementary file 1 [file ijms-26-04675-s001.zip › ijms-3538333-supplementary.pdf]

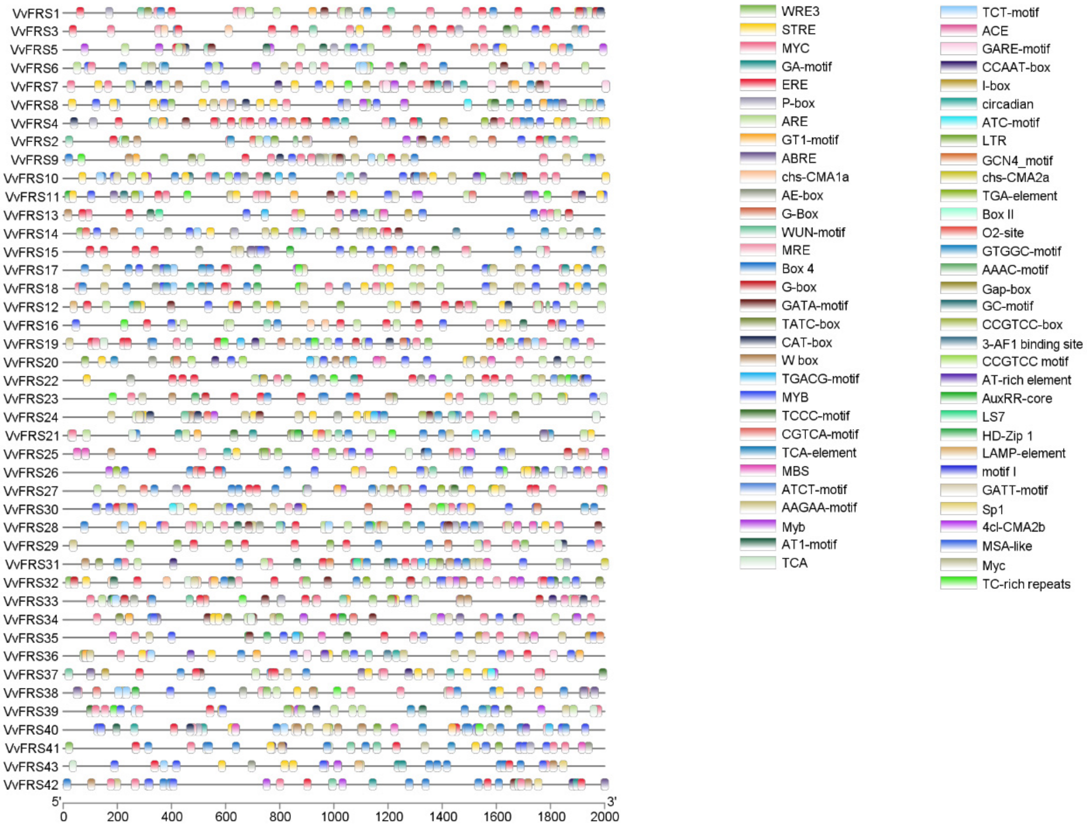

Figure S1. Schematic of cis-element in the promoter of *VvFRSs*.

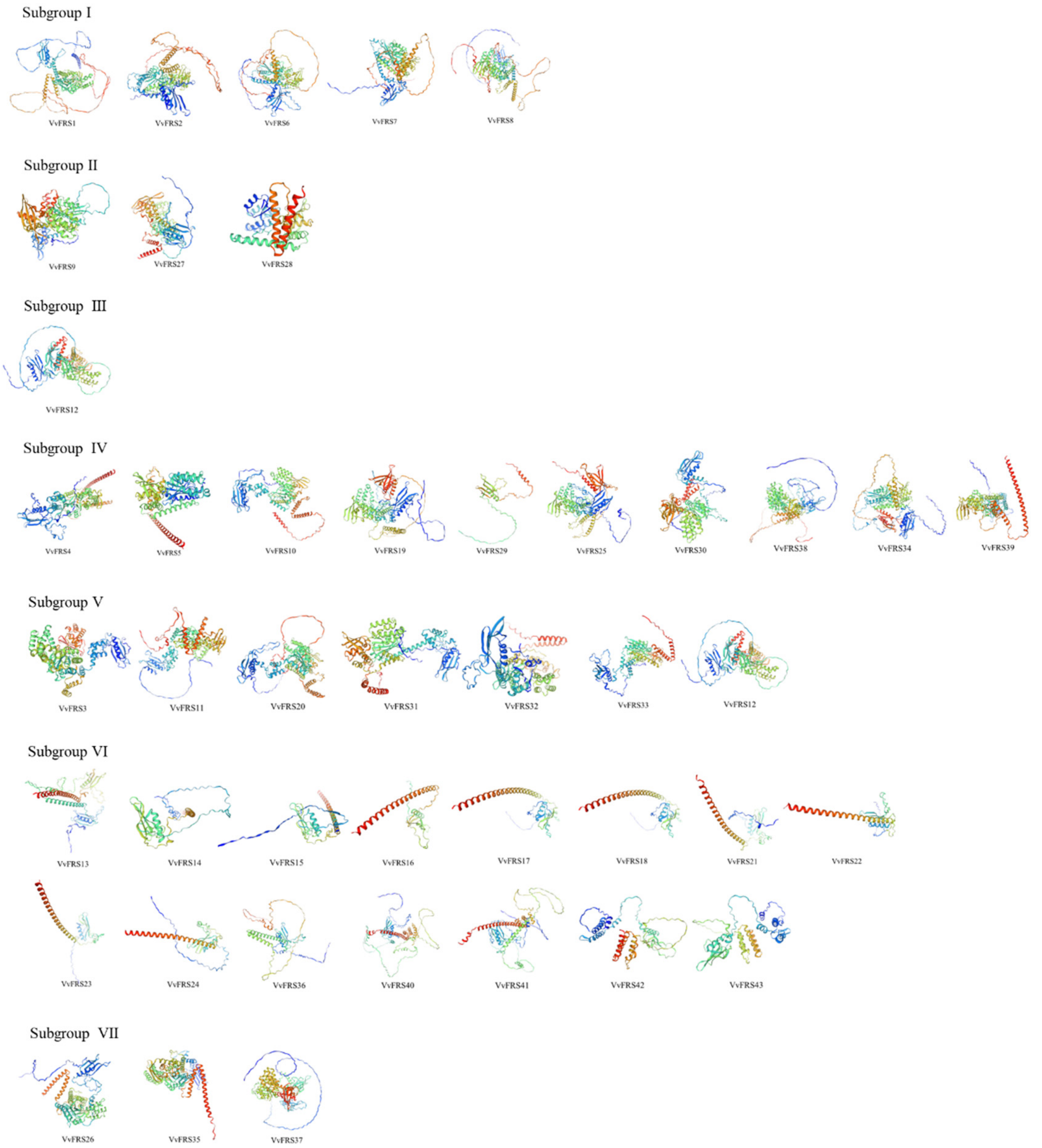

Figure S2. Three-dimensional models of VvFRS proteins. The color gradient from blue to red in the figure represented the sequential arrangement of protein structures from N-terminal to C-terminal.

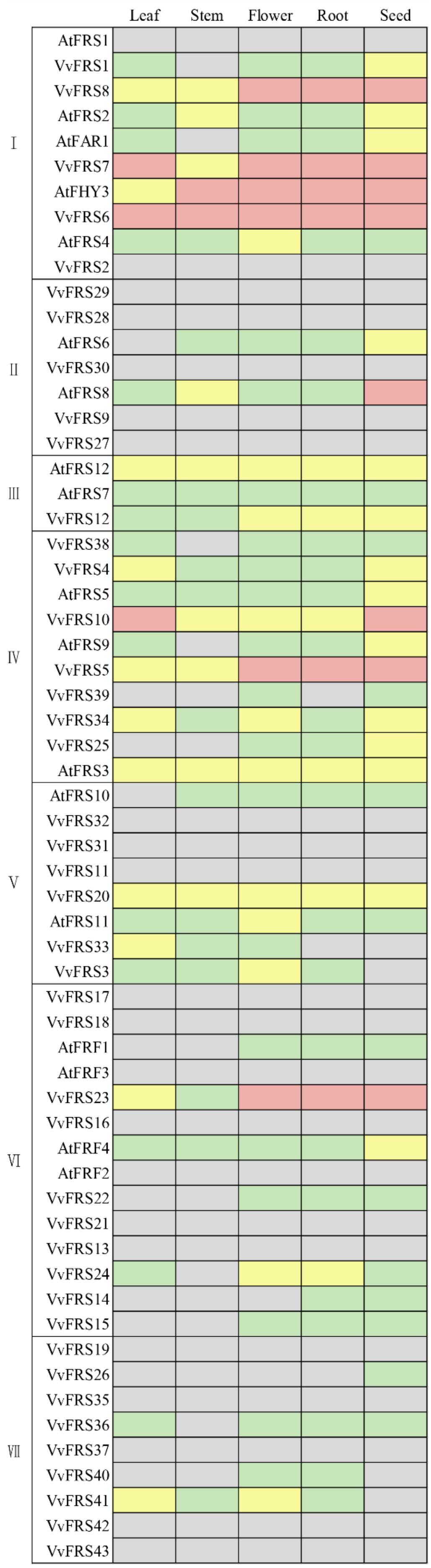

Leaf
Stem
Flower
Root
Seed
I
AtFRS1
VvFRS1
VvFRS8
AtFRS2
AtFAR1
VvFRS7
AtFHY3
VvFRS6
AtFRS4
VvFRS2
II
VvFRS29
VvFRS28
AtFRS6
VvFRS30
AtFRS8
VvFRS9
VvFRS27
III
AtFRS12
AtFRS7
VvFRS12
IV
VvFRS38
VvFRS4
AtFRS5
VvFRS10
AtFRS9
VvFRS5
VvFRS39
VvFRS34
VvFRS25
AtFRS3
V
AtFRS10
VvFRS32
VvFRS31
VvFRS11
VvFRS20
AtFRS11
VvFRS33
VvFRS3
VI
VvFRS17
VvFRS18
AtFRF1
AtFRF3
VvFRS23
VvFRS16
AtFRF4
AtFRF2
VvFRS22
VvFRS21
VvFRS13
VvFRS24
VvFRS14
VvFRS15
VII
VvFRS19
VvFRS26
VvFRS35
VvFRS36
VvFRS37
VvFRS40
VvFRS41
VvFRS42
VvFRS43

Figure S3. Comparison of expression levels between grape and Arabidopsis *VvFRS* genes in different organs.

Table S1. Ka/Ks values in synteny analysis

Seq_1	Seq_2	Ka	Ks	Ka/Ks	EffectiveLen	AverageS-sites	AverageN-sites	cN	cS	pN	pS	note
transcript:Solyc07g045520.3.1	transcript:Vitvi12g00713_t001	0.149453	1.009658	0.148023	2073	460.5	1612.5	218.5	255.5	0.135504	0.554832	VvFRS32
transcript:Solyc04g082845.1.1	transcript:Vitvi18g00039_t001	0.206901	1.728447	0.119704	2190	497.6667	1692.333	306	336	0.180815	0.675151	VvFRS39
transcript:AT4G19990.2	transcript:Vitvi01g00116_t001	0.314317	1.607278	0.195559	2268	472.9167	1795.083	460.9167	313.0833	0.256766	0.662026	VvFRS1
transcript:Solyc02g068550.2.1	transcript:Vitvi01g00116_t001	0.240478	1.459186	0.164803	2355	506.75	1848.25	380.25	325.75	0.205735	0.642822	VvFRS1
transcript:Solyc07g043260.3.1	transcript:Vitvi03g01239_t001	0.431011	1.396665	0.3086	2343	503.4167	1839.583	603.0833	318.9167	0.327837	0.633504	VvFRS6
transcript:Solyc06g065945.1.1	transcript:Vitvi04g00504_t001	0.219945	1.111972	0.197797	1128	244.0833	883.9167	168.5	141.5	0.190629	0.57972	VvFRS9
transcript:AT3G06250.2	transcript:Vitvi05g01873_t001	0.260715	NaN	NaN	2247	504.0833	1742.917	383.8333	396.1667	0.220225	0.785915	VvFRS12
transcript:AT5G18960.2	transcript:Vitvi05g01873_t001	0.260292	2.847608	0.091407	2259	505	1754	385.75	370.25	0.219926	0.733168	VvFRS12
transcript:AT2G43280.1	transcript:Vitvi05g04445_t001	0.387692	1.987465	0.195068	573	128.1667	444.8333	134.6667	89.33333	0.302735	0.697009	VvFRS16
transcript:Solyc06g073210.3.1	transcript:Vitvi05g01506_t001	0.387918	1.464815	0.264824	645	139.8333	505.1667	153	90	0.30287	0.643623	VvFRS17
transcript:AT3G59470.2	transcript:Vitvi05g01510_t001	0.404529	1.704764	0.237293	645	137	508	158.8333	92.16667	0.312664	0.672749	VvFRS18
transcript:AT2G27110.1	transcript:Vitvi06g01048_t001	0.185498	1.372528	0.135151	2541	572.5	1968.5	323.5	360.5	0.164338	0.629694	VvFRS19
transcript:Solyc09g075640.3.1	transcript:Vitvi07g00384_t001	0.136579	0.984775	0.13869	2271	501.75	1769.25	220.9167	275.0833	0.124865	0.548248	VvFRS20
transcript:AT1G52520.1	transcript:Vitvi09g00649_t001	0.399279	2.437671	0.163795	2046	453.5833	1592.417	493	327	0.309592	0.720926	VvFRS27
transcript:AT1G80010.1	transcript:Vitvi09g00649_t001	0.48311	NaN	NaN	2037	456.75	1580.25	562.8333	353.1667	0.356167	0.773217	VvFRS27
transcript:Solyc06g068210.2.1	transcript:Vitvi09g00649_t001	0.388962	1.552472	0.250544	2118	466.6667	1651.333	501.1667	305.8333	0.303492	0.655357	VvFRS27

Table S2. Primers used in RT-qPCR.

Primer name	Sequence（5' to 3'）
VvFRS43-F	CTCCTTTACAGCACCCCAGT
VvFRS43-R	GCACTCCAACAAATGGGCTT
VvFRS6-F	CTGAGCCAGAAGTTTTGGCT
VvFRS6-R	CCCTGAATTGTCTGCTGATT
VvFRS30-F	CGAAATCACTATGGAGGAAGGT
VvFRS30-R	TCAAAATCTGTGACATTTTCATG
VvFRS4-F	CAAATCTCCGGCCCTGCTAA
VvFRS4-R	AGCTGAATGTCGCCTTCCAA
VvFRS12-F	GCGTGAAAGTTGGGTTCCAC
VvFRS12-R	TTGTCTTTCCTCCTCACGGC
VvFRS35-F	TGAGTCACCAGATGGGTTGC
VvFRS35-R	TTCGTGACATCACACTGCCA
VvFRS14-F	GCCTATGGTAGGCGTACTGG
VvFRS14-R	TTCTCGGGTGATTGGTGGTG
VvFRS23-F	CACACGTCAAGGGTGCAAAG
VvFRS23-R	GATGCTCCACCTCCATGGTC
VvFRS5-F	CTTCCTGAGACCCGTCATCG
VvFRS5-R	TTCCAGGAGCGATTCCCAAC
VvFRS33-F	AGTTCGTTTCCTTCCTGCGT
VvFRS33-R	CCTCCGGATCCAGTTTCCTG
VvFRS7-F	TGAATGAAGAGCAGCGCCTA
VvFRS7-R	GTCTCATCCGCAATCAACGC
VvFRS18-F	GAGAAAGGCTGGTTGCAAGG
VvFRS18-R	CGTCTCTTCTCAAAGGCCAGT
VvFRS28-F	TCGTGCTGCATGTGGTTACT
VvFRS28-R	CTGATGTCTCACCCGCAAGT
VvFRS32-F	CCCCAGGGATCGGAAATCAG
VvFRS32-R	ATATGCAGGAAGCAGACGCA
